# Longitudinal comparison of the humoral immune response and viral load of Porcine Circovirus Type 2 in pigs with different vaccination schemes under field conditions

**DOI:** 10.12688/f1000research.13160.2

**Published:** 2018-08-29

**Authors:** Diana S. Vargas-Bermudez, Andrés Díaz, José Darío Mogollón, Jairo Jaime

**Affiliations:** 1 Departamento de Salud Animal. Facultad de Medicina Veterinaria y de Zootecnia, Universidad Nacional de Colombia, Bogotá, Colombia; 2 PIC LATAM, Mexico City, Mexico

**Keywords:** Porcine Circovirus type 2 (PCV2), PCV2 vaccines, IgG anti PCV2, viral loads.

## Abstract

**Background**: Porcine Circovirus type 2 (PCV2) infections are distributed worldwide and cause Porcine Circovirus Associated Disease (PCVAD). To minimize the impact of PCV2 infection on swine health and production, different vaccination schemes have been used since 2006. However, the association between vaccination schemes, virus load and disease under field conditions are not completely understood. Therefore, the objective of this study was to compare the effect of two different PCV2 vaccination schemes on the humoral response and PCV2 load in pigs after weaning under field conditions.

**Methods**: Two commercial pig farms (Farm A and B), endemically infected with PCV2, which were using two different PCV2 subunit vaccinations schemes for sow, gilts and piglets, were selected. We designed a longitudinal study and measured IgG levels by ELISA and virus load by quantitative PCR in pigs after weaning. Forty 3-week old piglets were randomly selected at weaning and followed for 20 weeks. IgG levels and virus loads were compared within and between farms and considered statistically different if the non-parametric Wilcoxon-test p value was lower than 0.05.

**Results**: We found that low virus loads were maintained in pigs from both farms regardless of the vaccination scheme used (p>0.05). However, there was significant difference in the mean IgG levels observed over time (p<0.05) while there were no significant differences in viral loads. This suggests that different humoral immune response is not associated with different virus loads observed over time.

**Conclusions**: These results are important because they can help to prevent PCV2 infections using different vaccination schemes to minimize the effect of PCVAD on swine health and production.

## Introduction

Porcine circovirus type 2 (PCV2) belongs to the *Circoviridae* family. It is a non-enveloped icosahedral virus with a single-stranded circular DNA genome that contains 1766 to 1768 nucleotides ( Fenaux *et al.*, 2004; Guo *et al.*, 2010). The PCV2 genome contains four open reading frames (ORFs), namely ORF1, ORF2, ORF3 and ORF4 ( Allan *et al.*, 2012; Xiao *et al.*, 2015). ORF1 encodes the Rep and Rep´ proteins required for viral replication, ORF2 encodes the immunogenic capsid protein (Cap) ( Fenaux *et al.*, 2004), ORF3 encodes a protein involved in apoptosis (ORF3 protein)( Liu *et al.*, 2005) and ORF4 encodes a protein that affects the activity of CD4+ and CD8+ cells ( He *et al.*, 2013). Additionally, the nucleotide diversity of ORF2 sequences allows to differentiate five different PCV2 genotypes denominated PCV2a, PCV2b, PCV2c, PCV2d (formerly known as mutant PCV2b) and PCV2e ( Davies *et al.*, 2016; Franzo *et al.*, 2015b; Xiao *et al.*, 2015). PCV2a and PCV2b are distributed worldwide, although PCV2b is more prevalent than PCV2a ( Opriessnig *et al.*, 2013). Until 2015, PCV2c was only reported in Denmark ( Dupont *et al.*, 2008); however, it is now reported in feral pigs in Brazil ( Franzo *et al.*, 2015a). Additionally, PCV2d is found in several countries, including China, Brazil, and USA ( Davies *et al.*, 2016; Franzo *et al.*, 2015a; Guo *et al.*, 2010; Xiao *et al.*, 2015; Zhai *et al.*, 2011). Moreover, the distant PCV2 genotype (PCV2e) is found in China ( Wang *et al.*, 2009) and the USA ( Davies *et al.*, 2016). In Colombia, PCV2 infections have been described since 2002 and have been recently characterized ( Rincón Monroy *et al.*, 2014).

Several syndromes collectively named Porcine Circovirus Associated Disease (PCVAD) are associated with PCV2 infections, and high PCV2 viral loads have been associated with disease severity ( Olvera *et al.*, 2004). PCVAD include PCV2-subclinical infection (PCV2-SI), PCV2 systemic disease (PCV2-SD, initially named as post-weaning multisystem wasting syndrome (PMWS), PCV2-reproductive disease (PCV2-RD), porcine dermatitis and nephropathy syndrome (PDNS), respiratory complex and enteritis ( Segalés, 2012; Shen *et al.*, 2010). PCV-SD is considered the most economically significant condition for the swine industry among all PCVAD ( Segalés, 2012).

PCVAD prevention is mainly based on vaccination against PCV2 infections ( Feng *et al.*, 2014; Fort *et al.*, 2009), this has led to a decrease in the prevalence of the virus and in the levels of viremia ( Dvorak *et al.*, 2016). PCV2 vaccination is effective in reducing viral load, viral shedding, and PCV2-SD associated lymphoid lesions ( Cline *et al.*, 2008; Fachinger *et al.*, 2008; Fort *et al.*, 2008; Park *et al.*, 2014). Vaccination can also induce neutralizing antibodies and IFNγ secreting cells (IFNγ SCs), which facilitates viral clearance ( Fort *et al.*, 2009; Martelli *et al.*, 2011). Additionally, PCV2 vaccination can minimize the effect of PCV2 infection on swine health improving average daily weight gain (ADWG) and reducing mortality, especially in the presence of co-infection with other viruses ( Fachinger *et al.*, 2008; Horlen *et al.*, 2008; Kixmöller *et al.*, 2008; Park *et al.*, 2014).

There are at least four different types of commercial PCV2 vaccines based on the PCV2a genotype worldwide ( Opriessnig *et al.*, 2014; Park *et al.*, 2014) that are effective at reducing the impact of PCV2a and PCV2b infections ( Fort *et al.*, 2008). One inactivated vaccine contains whole PCV2 as the antigen, and is recommend for 3-week old piglets or breeding females ( Beach & Meng, 2012; Segalés, 2015). In contrast, chimeric PCV1-2 vaccine contains the immunogenic capsid gene of PCV2a cloned into the genome backbone of the non-pathogenic PCV1 ( Segalés, 2015). Moreover, subunit recombinant vaccines express the capsid protein within a baculovirus system ( Shen *et al.*, 2010; Trible & Rowland, 2012) and are recommended for pigs between 2 and 4 weeks of age. However, off-label use of the chimeric vaccines in sows and gilts can result in the reduction of viremia and increased ADWG in the offspring ( Fraile *et al.*, 2012; Segalés, 2015). Vaccination of sows seeks to reduce viremia and viral loads in piglets through neutralizing antibodies present in colostrum, and could improve the productive performance of their offspring after weaning ( Beach & Meng, 2012; Gerber *et al.*, 2011; Pejsak *et al.*, 2010). Moreover, vaccination of the piglet is used to induce active humoral and cellular immunity, reduce viral loads, shorten duration of viremia, and improve productive performance ( Fachinger *et al.*, 2008; Fraile *et al.*, 2012; Lyoo *et al.*, 2011; Takahagi *et al.*, 2010). Currently, it is feasible to vaccinate sows, piglets, or both ( Fraile *et al.*, 2012; Opriessnig *et al.*, 2010), although the interference between maternally derived antibodies and active immunity of the piglet is under debate ( Fraile *et al.*, 2012).

Although it is well known that vaccination reduces the clinical presentation of the disease, limited information is available regarding the effect of different PCV2 vaccination schemes on virus load and humoral immune response over time under field conditions. Therefore, the objective of this study was to compare the effect of two different PCV2 vaccination schemes on the humoral response and PCV2 load in pigs after weaning. Our results indicated that different vaccination schemes against PCV2 induce different humoral immune responses overtime without a difference in the viral load observed. These results are important because they can help to prevent PCV2 infections and minimize the effect of PCVAD on swine health and production.

## Methods

### Farms and sample selection

For this study two commercial pig farms in Colombia (Farm A and B), endemically infected with PCV2, were conveniently selected. Farm A was 500-sow farrow-to-finish but with the nursery all in all out but it is close to site1; the site 3 is distant with continuous flow management. Farm B was 250-sow farrow-to-wean farm, with two additional sites for the nursery and finishing stages of production. Sites 1, 2 and 3 are geographically distant and the nursery or site two is all in all out; the site 3 is distant from site 2 but management is in continuous flow. Farm A vaccinated all sows and gilts (replacement animals for the breeding stock) against PCV2 every six months and all piglets on a weekly basis at 3 weeks of age. In contrast, Farm B vaccinated all gilts at arrival and piglets at 3 and 5 weeks of age on a weekly basis.

Forty 3-week old piglets were randomly selected at weaning in each farm. Each pig was ear tagged and randomly assigned to two treatments groups: non-vaccinated pigs (n=10) and PCV2 vaccinated pigs (n=30). Piglets with different treatments were comingled among other pigs after weaning based on the farmer’s production system. Animal care and procedures at the farms were in accordance with the guidelines of the "Porcine Animal Welfare" guide (Pork Colombia, former Colombian Association of Pig Farmers), which is based on the concept of the five freedoms (established by the Welfare Council of Farm Animals, 1992 in the United Kingdom). The pens are in cement with plastic Slat zones, water troughs with water ad libitum, feeders and a rest area in straw. The densities were managed according to the weight of the pigs following the recommendations of guideline 2008/120/EC. Pigs were injected intramuscularly on the right side of the neck at 3 weeks of age (weaning) with 1ml of commercial subunit vaccine A (VAC-A) in Farm A or 2ml of commercial subunit vaccine B (VAC-B) in Farm B. Additionally, pigs in Farm B were boosted with VAC-B at 5 weeks of age. Individual blood samples (10 ml) were collected by jugular venipuncture at 3, 7, 11, 15, 19 and 23 weeks of age (W3, W7, W11, W15, W19, and W23, respectively).

### ELISA and quantitative polymerase chain reaction (qPCR)

IgG antibodies against PCV2 were evaluated by ELISA using the INGEZIM Circo IgG1.1® assay (Ingenasa-Spain) at 450nm on a BioTek® Power Wave XS OD system with a cutoff value of 0.3, according to the manufacturer´s instructions.

Additionally, PCV2 viral loads were estimated over time using quantitative polymerase chain reaction (qPCR) ( Olvera *et al.*, 2004) in a Light Cycler® 480 II-Roche thermal cycling system. Briefly, DNA extractions were first performed from all serum samples collected using QIAamp DNA kit (QIAGEN®). Then PCV2 rep coding region of PCV2 was amplified using PCV2-ABF 5´GCCAGAATTCAACCTTMACYTTYC 3´ and PCV2-ABR 5´GCGGTGGACATGMTGAGATT 3´ primers, as previously described ( Rincón Monroy *et al.*, 2014). PCR reactions were carried out in 20µl containing 5μl of DNA mixed with 15μl of real-time PCR master mix (Light Cycler® 480 SYBR Green I Master-Roche mix + 1μM of each primer) at 95°C for 1 minute followed by 40 cycles of 95°C for 1 minute, 61°C for 25 seconds and 72°C for 5 seconds. Additionally, a plasmid (PCR blunt vector plasmid) containing the complete PCV2 genome was used as positive control (kindly donated by Dr. Carl A. Gagnon, Swine and poultry infectious diseases research center -CRIPA, Université de Montréal, St-Hyacinthe, Québec, Canada). Ten-fold dilutions of the plasmid (from 10 ^9 ^to 10 ^1^ PCV2 plasmid copies/ ml) were used as standard curve for PCV2 quantification. The cutoff level to diagnose animals as PMWS positive was established at 10 ^7 ^PCV2 genomes/ml, according to previous studies ( Olvera *et al.*, 2004). Piglets with viral loads lower than 10 ^7^ were considered asymptomatic animals ( Olvera *et al.*, 2004). Data analysis was done using the corresponding software (Light Cycler® 480 II-Roche).

### Statistical analysis

Mean IgG and PCV2 copies/ml were compared within and between VAC groups and considered statistically different if the non-parametric Wilcoxon-test p value was lower than 0.05. Additionally, the linear association between ELISA titters and the viral load was estimated at each sampling event and considered statistically significant if the null hypothesis of slope equal to 0 was rejected. The software used was R statistics version 3.4.1.

## Results

### Anti PCV2-IgG response

**Figure 1.**
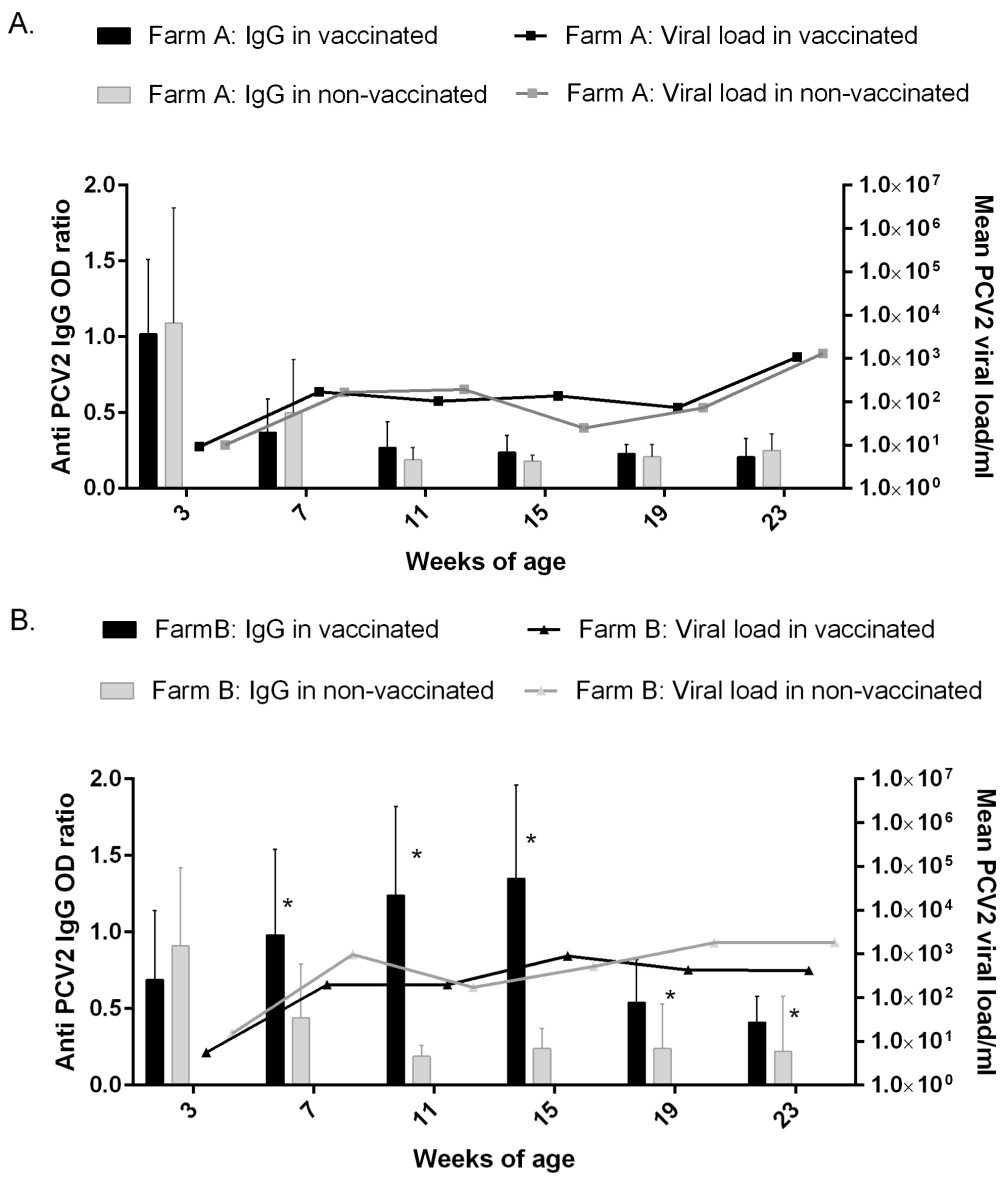
ELISA and PCV2 viral load comparison between vaccinated and non-vaccinated pigs in Farm **A** (panel **A**) and Farm **B** (panel **B**). Bars indicate the mean IgG level in vaccinated (black) and non-vaccinated (grey) pigs at 3, 7, 11, 15, 19, and 23 weeks of age. Lines indicate the mean PCV2 viral load in vaccinated (black) and non-vaccinated (grey) pigs at 3, 7, 11, 15, 19, and 23 weeks of age. *p<0.05.

All piglets had IgG antibodies against PCV2 at weaning and there was no statistical difference between treatment groups within farms before vaccination ( Table 1). However, at 3 weeks of age the anti-PCV2 IgG levels were higher in piglets from Farm A (VAC-A) than in piglets from Farm B (VAC-B) (p<0.05). The anti-PCV2 IgG response after vaccination was different between farms. In Farm A, IgG levels were high at 3 weeks of age and then decreased over time without significant difference in the average level of anti-PCV2 IgG between vaccinated and non-vaccinated pigs from Farm A (VAC-A) at each sampling event over time ( Table 1, p>0.05). Additionally, the mean optical density values obtained from pigs in Farm A overtime demonstrated that there was no seroconversion ( Figure 1A). Moreover, in Farm B IgG levels increased after vaccination until week 15 of age when they started to decrease, while non-vaccinated pigs from the same farm did not seroconvert ( Figure 1B) and showed statistically lower IgG titers over time (p<0.05, Table 1) compared to vaccinated pigs within the same farm. Interestingly, none of the vaccinated or non-vaccinated pigs in this study had anti-PCV2 IgG levels greater than 0.41 after 23 weeks of age.

**Table 1.** Mean IgG levels distributed by week of age (3, 7, 11, 15, 19, and 23), farm (A and B), and treatment (vaccinated and non-vaccinated). Mean IgG levels are compared within farm (vaccinated vs. non-vaccinated) and between farms (VAC-A vs. VAC-B). Different letters within farm indicate a significant difference (p<0.05) in the mean IgG level between vaccinated and non-vaccinated pigs. The significance level of difference in the IgG level between vaccinated pigs in farm A (VAC-A) and Farm B (VAC-B) by week are indicated with * (p<0.05) and ** (p<0.01). Pigs were vaccinated at week 3 and on farm B they received a booster at week 5.

Sampling - Weeks of age	Farm A				Farm B			
	Vaccinated		Non-vaccinated		Vaccinated		Non-vaccinated	
	IgG mean	Sd	IgG mean	Sd	IgG mean	Sd	IgG mean	Sd
3 *	1.02 ^a^	0.49	1.09 ^a^	0.76	0.69 ^a^	0.45	0.91 ^a^	0.51
7 **	0.37 ^a^	0.22	0.50 ^a^	0.35	0.98 ^a^	0.56	0.44 ^b^	0.35
11 **	0.27 ^a^	0.17	0.19 ^a^	0.08	1.24 ^a^	0.58	0.19 ^b^	0.07
15 **	0.24 ^a^	0.11	0.18 ^a^	0.04	1.35 ^a^	0.61	0.24 ^b^	0.13
19 **	0.23 ^a^	0.06	0.21 ^a^	0.08	0.54 ^a^	0.28	0.24 ^b^	0.29
23 **	0.21 ^a^	0.12	0.25 ^a^	0.11	0.41 ^a^	0.17	0.22 ^b^	0.36

Sd: Standard deviation

### PCV2 viral loads

 All serum samples from this study tested PCR positive for PCV2; however, none had a viral load greater than 10 ^4^ DNA copies/ml ( Figure 1A and B). Hence, all pigs were considered PCR positive, but with low viral loads, and therefore PMWS negative or asymptomatic during the study period. Additionally, there was no difference within farm in the viral load between vaccinated and non-vaccinated pigs and there was no difference found in the viral load between vaccinated pigs in Farm A (VAC-A) and vaccinated pigs in Farm B (VAC-B).

Data of the results obtained in the study The data obtained and analysed for the ELISA and qPCRy tests are available in an attached document where are classified by farms. Likewise, the results of the negative controls used are included. Click here for additional data file. Copyright: © 2018 Vargas-Bermudez DS et al. 2018 Data associated with the article are available under the terms of the Creative Commons Zero "No rights reserved" data waiver (CC0 1.0 Public domain dedication).

## Discussion

To better understand the effect of PCV2 vaccination on the IgG response and PCV2 viral loads in pigs after weaning, we designed a longitudinal study and compared two different vaccination schemes under field conditions. We found that the PCV2 viral load in pigs after weaning was not associated to the vaccine scheme used in each farm studied. However, we found differences in the IgG levels between farms that could be associated with vaccination schemes. Understanding the effect of different vaccines and vaccine schemes on virus load and humoral response is important to design better health intervention to control PCV2 infection and minimize its effect on swine health and production.

PCV2 vaccination has proven to control the effect of PCV2 infection on swine health and production ( Cline *et al.*, 2008; Horlen *et al.*, 2008; Kixmöller *et al.*, 2008) and there are different PCV2 vaccination schemes used in the contemporary swine industry. However, new PCV2 genotypes have been discovered ( Davies *et al.*, 2016; Xiao *et al.*, 2015) and vaccine failure has been described ( Fraile *et al.*, 2015; Wang *et al.*, 2009). In this study, we found low viral loads regardless of the vaccination scheme used in the farms studied. These findings were expected because vaccination can reduce the percentage of infectious pigs and the level of viremia in them ( Cline *et al.*, 2008; Dvorak *et al.*, 2016; Fachinger *et al.*, 2008; Feng *et al.*, 2014; Opriessnig *et al.*, 2010). It is possible that viral loads remained low due to continuous vaccination of the herd regardless of the vaccination scheme. It was interesting to find that non-vaccinated animals maintained low viral loads within farms endemically infected with PCV2. We speculate that finding non-vaccinated pigs with low viral titers was the result of the overall herd immunity. This is in agreement with the findings by Feng *et al.* ( Feng *et al.*, 2014), in which mass vaccination against PCV2 reduced viral loads at the population level. The presence of low viral loads in both vaccinated and unvaccinated pigs shows that the virus is circulating. Studies have shown that PCV2 is very stable in the environment, causing numerous routes of infection and that piglets can also be infected in the presence of maternal immunity ( Dvorak *et al.*, 2013)

In this study, we found differences in the humoral response between vaccinated pigs from Farm A and Farm B over time. This result can probably be explained by the second dose (booster) used in piglets in Farm B, although this cannot be concluded from the results obtained since the levels of neutralizing antibodies were not evaluated. Studies show that vaccination against PCV2 does not necessarily stimulate capsid-specific antibodies but does seem to be involved in the increase of neutralizing antibodies ( Dvorak *et al.*, 2018). In our study, vaccination against PCV2 using two doses in piglets results in a higher antibody response than a single dose (p<0.05), even though in terms of protection the two options have shown to be effective and control PCV2 viremia ( Lyoo *et al.*, 2011). However, a single dose at 3 weeks of age might interfere with maternal antibodies as described before ( Fort *et al.*, 2009; Fraile *et al.*, 2012; Martelli *et al.*, 2011). In our study, pigs from Farm A showed higher levels of maternal derived antibodies at weaning, did not seroconvert after a single vaccination, and showed low PCV2 loads over time. Pesjak *et al.* ( Pejsak *et al.*, 2010) and Opriessnig *et al.* ( Opriessnig *et al.*, 2010), demonstrated that the presence of maternal-derived antibodies do not affect the efficacy of PCV2 subunit vaccines and proved low concentrations of viral DNA in serum after vaccination (as seen in our study), absence of histological lesions, and improvement in the productive parameters. Moreover, the different humoral immune response between vaccinated and non-vaccinated pigs in Farm B corresponded to a classical pattern of antibody response due to vaccination. Furthermore, it is the classical profile of humoral response after weaning without virus circulating. The humoral immune profile of piglets and sows is determined by PCV2 circulation, vaccination schemes, and is associated with virus load in pigs after weaning. 

Fraile *et al.* ( Fraile *et al.*, 2015) defined four clusters of pigs based on PCV2 serological and PCR profiles. Cluster 1 is composed mainly by none vaccinated sows and none vaccinated pigs, in which viremic pigs are present with increasing antibody levels over time. Cluster 2 contains mostly vaccinated sows and non-vaccinated piglets in which late PCV2 infection and seroconversion is observed. Cluster 3 has mainly vaccinated sows and vaccinated pigs, viremia is rare and antibodies decrease over time; and cluster 4 is composed basically of non-vaccinated sows and vaccinated pigs in which infected animals are rare and high immununoperoxidase monolayer assay (IPMA) titers are observed. Regardless of the vaccination scheme used in our study (Farm A versus B) all pigs met the criteria of cluster 3, rare viremia and antibody induction over time, even though not all sows were vaccinated (Farm B).

The present study contributes to the understanding of PCV2 infection and control under field conditions. However, it is important to keep in mind that we assumed that farms were endemic infected with PCV2 although high viral loads were never observed. Therefore, we could not test if there was an appropriate protection induced by the vaccines or minimal virus challenge. Additionally, our low sample size for the non-vaccinated control groups (n=10) might had been insufficient to detect viremic pigs under very low prevalence of the virus at the population level.

Vaccination is a key intervention to control the impact of PCV2 on swine health and production. Our findings illustrated that, regardless of the vaccination scheme used, low viral loads of PCV2 were maintained, although a similar response was found in the unvaccinated group. This could indicate that when a farm has a vaccination program established some time ago, it can contribute to the control of the virus. This can probably be explained by the presence of neutralizing antibodies in the control group that were not detected by the ELISA test. These results are important because they can help to prevent PCV2 infections and minimize the effect of PCVAD on swine health and production. Future studies are required to understand the epidemiology of PCV2 infection in positive farms with very low prevalence of PCV2 infections.

## Ethical statement

The farms included in the study are associated with Pork Colombia and follow the guidelines of production, biosecurity and animal welfare required by this institution. Approval was requested from the farms where the study was conducted and they agreed to its completion. The veterinarians of each farm supervised and collaborated with the study. The Bioethics Committee of the Faculty of Veterinary Medicine and Animal Sciences of the National University of Colombia approved the procedures performed on the pigs (resolution OF-CBE-FMVZ-0006-10).

Every effort was made to reduce the suffering of the pigs to a minimum. Veterinarians trained in this procedure took the blood samples and the pigs were monitored for one hour after taking the sample to control for any adverse effects on the procedure.

## Data availability

The data referenced by this article are under copyright with the following copyright statement: Copyright: © 2018 Vargas-Bermudez DS et al.

Data associated with the article are available under the terms of the Creative Commons Zero "No rights reserved" data waiver (CC0 1.0 Public domain dedication). 

**Dataset 1: Data of the results obtained in the study.** The data obtained and analysed for the ELISA and qPCRy tests are available in an attached document where are classified by farms. Likewise, the results of the negative controls used are included. DOI, 10.5256/f1000research.13160.d188246 (Vargas-Bermudez *et al.*, 2017).
