# Supplementary material for: Longitudinal comparison of the humoral immune response and viral load of Porcine Circovirus Type 2 in pigs with different vaccination schemes under field conditions
Source: F1000Res. 2018 Aug 29;7:42. Originally published 2018 Jan 10. [Version 2] doi: 10.12688/f1000research.13160.2 (PMC6127736; doi:10.12688/f1000research.13160.2)
Supplement: Peer review history

# Peer review: Referee response for version 2

**DOI:** 10.5256/f1000research.17540.r37654

**Published:** 2018-09-24

**Authors:** Alvaro Rafael Ruiz-Garrido

**Affiliations:** 1 Facultad de Cs. Veterinarias, Universidad de Concepcion, Concepción, Chile

Regarding to the authors answer in 2.3.2, did not clarified the meaning of the * in the legend of the figure 1, as they say.

I have read this submission. I believe that I have an appropriate level of expertise to confirm that it is of an acceptable scientific standard.

# Peer review: Referee response for version 2

**DOI:** 10.5256/f1000research.17540.r37656

**Published:** 2018-09-06

**Authors:** Jesús Hernandez

**Affiliations:** 1 Laboratory of Immunology, Research Center for Food and Development (CIAD), Hermosillo, Mexico

I have no comments to make.

I have read this submission. I believe that I have an appropriate level of expertise to confirm that it is of an acceptable scientific standard.

# Peer review: Referee response for version 1

**DOI:** 10.5256/f1000research.14275.r30290

**Published:** 2018-02-13

**Authors:** Jesús Hernandez

**Affiliations:** 1 Laboratory of Immunology, Research Center for Food and Development (CIAD), Hermosillo, Mexico

The manuscript by Vargas-Bermudez *et al. *evaluated humoral response (IgG) and viremia in pigs from farms with two different vaccination programs. The authors concluded that viral loads were low regardless the vaccination schemes. The manuscript is well written, clear and provides interesting information.

**Major concerns:**

 Conclusions

 There are statements that are not supported by the results and have to be modified.

  

- “Another explanation for vaccinated and non-vaccinated pigs with low viral loads is that there was no PCV2 circulating in the farm and that continuous vaccination of the populations has indeed minimized PCV2 infection between pigs”.
  Positive PCR indicate that PCV2 is circulating in the farm.
- “Our findings illustrated that different vaccination schemes against PCV2 can maintain low viral load in endemically infected populations regardless of the different humoral immune profiles observed over time”.
  This statement is not correct, because non-vaccinated group maintained low viral loads.

 

**Minor concerns:**

 Results

- Can you include the IgG values of week 5?
- It is not clear the weaning age. It is week 3?
- The inclusion of vaccination time in the table 1 could help in the interpretation.

I have read this submission. I believe that I have an appropriate level of expertise to confirm that it is of an acceptable scientific standard, however I have significant reservations, as outlined above.

# Response

**Published:** 2018-08-20

**Authors:** Jairo Jaime

**Affiliations:** Universidad Nacional de Colombia

3. Jesus Hernandez:

 3.1. Discussion.

 3.1.1. In discussion, paragraph 2, you are correct in stating that: Another explanation for vaccinated and non-vaccinated pigs with low viral loads is that there was no PCV2 circulating in the farm and that continuous vaccination of the populations have indeed minimized PCV2 infection between pigs.

 This correction coincides with 1.3.1. Mike Murtaughg

 1.3.1. In Paragraph 2, you are right in stating that: Another explanation for vaccinated and non-vaccinated pigs with low viral loads is that there was no PCV2 circulating in the farm and that continuous vaccination of the populations has indeed minimized PCV2 infection between pigs. Evidently there was PCV2 circulation. The phrase was rectified and changed by: The presence of low viral loads in both vaccinated and unvaccinated pigs shows that the virus is circulating. Studies have shown that PCV2 is very stable in the environment, causing numerous routes of infection and that piglets can also be infected in the presence of maternal immunity (Dvorak et al., 2013).

 3.1.2. In discussion, paragraph 6 states: Our findings illustrated that different vaccination schemes against PCV2 can maintain low viral load in endemically infected populations, regardless of the different humoral immune profiles observed over time.

  This correction coincides with 2.2.7. (Alvaro Ruíz)

 2.2.7. Paragraph 6 states: Our findings illustrated that different vaccination schemes against PCV2 can maintain low viral load in endemically infected populations, regardless of the different humoral immune profiles observed over time. This statement is not correct since the unvaccinated group maintained low viral loads. This statement was corrected as follows:

 Our findings illustrated that, regardless of the vaccination scheme used, low viral loads of PCV2 were maintained, although a similar response was found in the unvaccinated group. This could indicate that when a farm has a vaccination program established some time ago, it can contribute to the control of the virus. This can probably be explained by the presence of neutralizing antibodies in the control group that were not detected by the ELISA test.

 3.2. Results:

 3.2.1. The IgG values can be included in week 5?

 It was not evaluated in that week (which was when the booster was made (second dose) in farm B.

 3.2.2. It is not clear the week of weaning, was week 3?

 Yes it was in week 3, this was added in the text in methods (paragraph 2).

 3.2.3. The inclusion of the vaccination time in table 1 could help the interpretation.

 This recommendation is shared and included in the text of the table.

# Peer review: Referee response for version 1

**DOI:** 10.5256/f1000research.14275.r30258

**Published:** 2018-02-09

**Authors:** Michael P. Murtaugh, Cheryl Dvorak

**Affiliations:** 1 Department of Veterinary and Biomedical Sciences, College of Veterinary Medicine, University of Minnesota, Saint Paul, MN, USA

The authors evaluated the effects of moderately different vaccination schemes on PCV2, concluding there was no substantial difference in the various vaccination schemes. The study is narrow in scope, having used two independent farms for the main treatment difference, and a different vaccine on each farm. These confounding factors limit the generalizability of the findings, which the authors are aware of. It is a common limitation in field studies that is a bit compensated for by the direct applicability of the findings. The lack of replication and the small sample sized are additional limitations that the authors acknowledge.

      It was a bit surprising that the sow herds were either fully vaccinated or entering gilts were vaccinated. This practice is to my knowledge uncommon in North America since it is obvious that capsid vaccines used only in the farrowing room and maybe the nursery are highly effective in preventing PCVAD in growing pigs. It also is common knowledge that the level of PCV2 viremia in finishing pigs has been reduced tremendously since vaccines were introduced in North America in 2006 (Dvorak et al. National reduction in porcine circovirus type 2 prevalence following introduction of vaccination. Vet Micro 189 [2016] 86-90). For this reason, much of the preceding published literature may be out of date and not relevant to the present situation.

      There is a wide variation in quality in the published literature that the authors might want to evaluate. Phylogenetics studies with hundreds or thousands of sequences (e.g. Davies. [2016] Diagnostic phylogenetics reveals a new porcine circovirus 2 cluster. Virus Res. 217:32-37) are superior to reports with tens of sequences which often miss important variants due to random chance. The authors also should refer to original and primary reports rather than reviews in citing new discoveries. 

 Minor comments:

 Methods. Farms and sample selection, 1 ^st^ paragraph – The phrase "vaccinated on a weekly basis" is confusing. Perhaps the phrase could be deleted or the vaccination scheme explained a bit more.

 Discussion. It is incorrect to say that no PCV2 was circulating in the farm, since there were PCV2 positive animals in all treatment groups. It also is clear that PCV2 is environmentally stable, providing for numerous sources of infection in the environment of the pigs (Dvorak et al. [2013] Multiple routes of porcine circovirus type 2 transmission to piglets in the presence of maternal immunity. Vet. Microbiol. 166:365-374.)

 Discussion. Third paragraph – “In this study, we found differences in the humoral response between vaccinated pigs from Farm A and Farm B over time, mainly explained by the second dose (booster) used in piglets in Farm B and the vaccination schemes used in gilts and the sows.” Because of the confounding factors present in the experimental design it really is not possible to conclude that the booster was the reason for the differences in humoral response.

 Interestingly, it has been shown previously that vaccination does not necessarily boost capsid-specific antibodies, but does seem to be involved in increasing neutralizing antibody titers. Perhaps, this is dependent upon the vaccine used, which would agree your data. (Dvorak et al. Effect of Maternal Antibody Transfer on Antibody Dynamics and Control of Porcine Circovirus Type 2 Infection in Offspring. Viral Immunology [2018] 31: 40-46.)

 Discussion. Fourth paragraph – “Fraile et al….”I think you mean non-vaccinated not “none vaccinated”.

We have read this submission. We believe that we have an appropriate level of expertise to confirm that it is of an acceptable scientific standard, however we have significant reservations, as outlined above.

# Response

**Published:** 2018-08-20

**Authors:** Jairo Jaime

**Affiliations:** Universidad Nacional de Colombia

1. Mike Murtaughg

 1.1. Introduction:

 1.1.1. It has the reason that it has been shown that the level of viremia has decreased in North America using vaccination, particularly in the United States. Correction was made, thus leaving the phrase: PCVAD prevention is mainly based on vaccination against PCV2 infections (Fort et al., 2009; Feng et al., 2014), which has led to a decrease in the prevalence of the virus and viremia levels (Dvorak et al., 2016).

 1.1.2. It has the reason that phylogeny should be compared with papers that support analysis of hundreds or thousands of sequences to obtain clearer clusters. In this case, the bibliography was corrected in the first paragraph of the introduction.

 1.2. Methods:

 1.2.1. The phrase ... at 3 and 5 weeks of age on a weekly basis. It was eliminated *on a weekly basis.*

 1.3. Discussion:

 1.3.1. In Paragraph 2, you are right in stating that: Another explanation for vaccinated and non-vaccinated pigs with low viral loads is that there was no PCV2 circulating in the farm and that continuous vaccination of the populations has indeed minimized PCV2 infection between pigs. Evidently there was PCV2 circulation. The phrase was rectified and changed by: *The presence of low viral loads in both vaccinated and unvaccinated pigs shows that the virus is circulating. Studies have shown that PCV2 is very stable in the environment, causing numerous routes of infection and that piglets can also be infected in the presence of maternal immunity (Dvorak et al., 2013).*

 1.3.2. In paragraph 3, you are right that it cannot be said that: In this study, we found differences in the humoral response between vaccinated pigs from Farm A and Farm B over time mainly explained by the second dose (booster) used in piglets in farm B and the vaccination schemes used in gilts and sows. The sentence was rectified as follows: *In this study, we found differences in the humoral response between vaccinated pigs from Farm A and Farm B over time. This result can probably be explained by the second dose (booster) used in piglets in Farm B, although this cannot be concluded from the results obtained since the levels of neutralizing antibodies were not evaluated. Studies show that vaccination against PCV2 does not necessarily stimulate capsid-specific antibodies but does seem to be involved in the increase of neutralizing antibodies (Dvorak et al., 2018).*

# Peer review: Referee response for version 1

**DOI:** 10.5256/f1000research.14275.r30289

**Published:** 2018-02-08

**Authors:** Alvaro Rafael Ruiz-Garrido

**Affiliations:** 1 Facultad de Cs. Veterinarias, Universidad de Concepcion, Concepción, Chile

I think that the paper is very good, and should be published, but there are somethings that must be improved before, as follows:

 •          Abstract

 The authors should review the conclusions, because they did not observe statistical differences between the vaccinated and unvaccinated groups, in both farms, regarding to the viral loads. They just studied 2 variables (humoral response and viral load) and they only found that the humoral response is different depending of the vaccine used.

 •          Methods

 The authors should explain better the differences that exist between both farms, from the infrastructural, animal’s flow and management point of view.

 It is lamentable that the authors do not have a replicate of the treatment in each farm, in order to give more power to the results.

 •          Statistical analysis

 The authors should use a test of repeated samples ANOVA, if the assumptions allow it.

 •          Results

 The authors should review the tables, because there are some differences in the results that are possible to obtain from the original data and the ones showed in tables (mean and standard deviation) and figures (mean) of the paper.

 In Figure 1, the authors should use the same scale for the ELISA results of farm A and B. Additionally, they have to indicate if the * indicate statistical differences between vaccinated and unvaccinated animals of farm B or statistical differences between the different weeks of sampling of vaccinated animals of farm B.

 •          Discussion

 The authors should discuss the phrase “However, at 3 weeks of age the anti-PCV2 IgG levels were higher in piglets from Farm A (VAC-A) than in piglets from Farm B (VAC-B) (p<0.05).” that it is in the results, and the implication that the farms were different from the beginning, and how this can influence the results that they obtain.

 As Figure 1 shows, the authors should give explanations for the serology decay of the vaccinated animals in farm B at the 19 and 23 weeks. Additionally, the authors should discuses why there is an increase, no statistical significance, in 3 of the 4 groups, at weeks 23, of the mean PCV2 DNA load, as show by Figure 1.

 There is a little of over conclusions, since it states that “In our study, vaccination against PCV2 using two doses in pig- lets results in a higher antibody response than a single dose (p<0.05), even though in **terms of protection the two options have shown to be effective and control PCV2 viremia**”; the authors cannot affirm this because there were no differences in viral load between control (un vaccinated) and vaccinated group.

 The authors should indicate what is the meaning of IPM in the clusters 4.

 The authors should review the sentence “Our findings illustrated that different vaccination schemes against PCV2 can maintain low viral load in endemically infected populations regardless of the different humoral immune profiles observed over time". The authors did not observe statistic differences between the control group and the vaccinated group, so they can not affirm the above sentence.

 It could have been interesting if the authors had measured the productive parameters of the treatment groups, to see any difference, but with the reduced number of animals (especially the control group) this was not possible.

 The authors should discus the differences between farms (including the Ig G level at the beginning of the essay), vaccine used, vaccine protocols and epidemiology of PCV2 at the population level and there effect on they results.

I have read this submission. I believe that I have an appropriate level of expertise to confirm that it is of an acceptable scientific standard, however I have significant reservations, as outlined above.

# Response

**Published:** 2018-08-20

**Authors:** Jairo Jaime

**Affiliations:** Universidad Nacional de Colombia

2. Alvaro Ruíz Garrido

 2.1. Abstract

 2.1.1. Include in the abstract the variables handled (humoral response and viral load) indicating that there are only changes in the immune response.

 In the Abstract, the paragraph of methods says what the reviewer is requesting: We designed a longitudinal study and measured IgG levels by ELISA and virus load by quantitative PCR in pigs after weaning. In the abstract also, in the paragraph of results, the following sentence was added: We found that low virus loads were maintained in pigs from both farms, regardless of the vaccination scheme used (p> 0.05). However, IgG levels were observed over time (p <0.05) *while no significant differences were found in viral loads. This suggests that different humoral immune response is not associated with different virus loads observed over time.*

 2.2. Methods:

 2.2.1. Indicate the differences between farms in terms of infrastructure, animal flow and management. The sentence was rectified as follows:

*For this study two commercial pig farms in Colombia (Farm A and B), endemically infected with PCV2, were conveniently selected. Farm A was 500-sow farrow-to-finish but with the nursery all in all out but it is close to site1 and site 3 is distant with continuous flow management. Farm B was 250-sow farrow-to-wean farm, with two additional sites for the nursery and finishing stages of production. Sites 1, 2 and 3 are geographically distant and the nursery or site two is all in all out. Site three is distant from site two but management is in continuous flow.*

 2.2.2. In the statistical analysis because an ANOVA test of repeated samples was not done.

 We decided to not use ANOVA because the data was not normally distributed and it was not independent at all (vaccinated and not vaccinated animals were in the same farm). Hence we used a non parametric test to compare the mean of to correlated samples.

 2.3. Results

 2.3.1. Review the tables since there is a difference of results between the original data and those of the tables (SD / media)

 The reviewer is correct and the mean and SD for animals in farm A in Table 2 are incorrect. The table has been updated with the right values for each group. However the statistical difference noted within and between farms are correct.

 2.3.2. In figure 1 use the same scale for the ELISA. The * indicates differences between vaccinates (farm B) or statistical differences over time.

 In Figure 1, different scales were used so that the reader can better visualize the differences, however it is considered that the reviewer is right and the two graphs with the same scale were adjusted. The * indicates difference between vaccinated and unvaccinated in each evaluated week, not in time. This is clarified in the legend of the figure.

 2.4.3. In paragraph 2, the sentence: However, at 3 weeks of age the anti-PCV2 IgG levels were higher in piglets from Farm A (VAC-A) than in piglets from Farm B (VAC-B) (p <0.05). What implications does this have from the beginning and how can it influence the results?

 The presence of higher levels of antibodies in farm A both in vaccinated and unvaccinated pigs at the beginning of the experiment (week 3) could be indicating that there was a greater transmission of maternal antibodies compared to farm B, without any of the farms this difference will affect the viral load.

 2.2.4. In Figure 1. Why there is a decline in the serology of vaccinated pigs in farm B at 19 and 23 weeks. It should be analyzed why there is an increase, without statistical significance, in 3 of the 4 groups in week 23 of the average PCV2 DNA load?

 The decay of antibodies at weeks 19 and 23 in farm B in the vaccinated group without modification of the viral load would indicate that the antibodies detectable by ELISA have been metabolized, but do not show that the pigs have lost protection. By week 23, an increase in viral loads (without statistical significance) could be established, which could indicate that viral loads were increasing while antibodies were decreasing and if the pigs were kept longer it is likely that viral loads increased to levels of risk. (This is better in the discussion)

 2.3. Discussion

 2.3.1. In paragraph 3 it cannot be stated: In this study, we found differences in the humoral response between vaccinated pigs from Farm A and Farm B over time mainly explained by the second dose (booster) used in piglets in farm B and the vaccination schemes used in gilts and sows.

 This correction coincides with 1.3.2. Mike Murtaughg

 1.3.2. In paragraph 3, you are right that it cannot be said that: In this study, we found differences in the humoral response between vaccinated pigs from Farm A and Farm B over time mainly explained by the second dose (booster) used in piglets in farm B and the vaccination schemes used in gilts and sows. The sentence was rectified as follows: In this study, we found differences in the humoral response between vaccinated pigs from Farm A and Farm B over time. This result can probably be explained by the second dose (booster) used in piglets in Farm B, although this cannot be concluded from the results obtained since the levels of neutralizing antibodies were not evaluated. Studies show that vaccination against PCV2 does not necessarily stimulate capsid-specific antibodies but does seem to be involved in the increase of neutralizing antibodies (Dvorak et al., 2018).

 2.3.2. What does IPM mean?

 It was corrected by IMPA (immunoperoxidase monolayer assay)

 2.2.7. Paragraph 6 states: Our findings illustrated that different vaccination schemes against PCV2 can maintain low viral load in endemically infected populations, regardless of the different humoral immune profiles observed over time. This statement is not correct since the unvaccinated group maintained low viral loads. This statement was corrected as follows:

*Our findings illustrated that, regardless of the vaccination scheme used, low viral loads of PCV2 were maintained, although a similar response was found in the unvaccinated group. This could indicate that when a farm has a vaccination program established some time ago, it can contribute to the control of the virus. This can probably be explained by the presence of neutralizing antibodies in the control group that were not detected by the ELISA test.*

 2.3.3. The authors should discuss the differences between farms (including the IgG level at the beginning of the trial), the vaccine used, the vaccine protocols and the epidemiology of PCV2 at the population level and its effect on the results.

 It is likely that the level of neutralizing antibodies in both farms was sufficient to control the virus. The implication of these initial levels of antibodies in the results is not clear and could be on the response of the same. In farm A, it was able to influence the levels of antibodies to remain low throughout the experiment, while in farm B he was able to increase them. The foregoing is explained by the consumption of the antibodies against the vaccine challenge at 3 weeks (farm A).
